# Hospital Questions Which Press

**Published:** 1905-02-25

**Authors:** 


### HOSPITAL QUESTIONS WHICH PRESS.

### WANT OF CO-OPERATION.

(*Continued from Page* 375.)

CHARITY COMPETITIONS.

VALUABLE as the independent efforts of the hospitals have been in the past to obtain the money requisite for their needs by a succession of appeals to the metropolis as a whole, it is growing more demonstrable every year that this method has had its day. The more this matter is considered in all its bearings the stronger does the conviction become that each hospital in its endeavours to obtain public support ought to concentrate its energies upon the locality which it especially serves and confine its efforts to that locality alone. The evils of individual attempts to obtain subscriptions from the entire public are now tending to outweigh all the good that may be achieved. By imperceptible steps, owing to the habit that has grown of each hospital confining its attention solely to its own needs, these institutions have been drawn into taking part in what is gradually becoming nothing less than a most objectionable competition for public charity. It seems now to be almost a question of which one can make the most pitiable appeal. Some of the managers, in making their special supplications, have altogether left the realms of common sense and adopted a species of pathetic humbug which is nothing short of ludicrous. The man in the street confesses his amusement; and worse than this there appears to be a possibility that such highly coloured epistles may ultimately have the effect of convincing a small but important section of the public that some of the London voluntary hospitals are extremely artful beggars who make a point of being a little in debt for the sake of the extra pathos which they are thus able to instil into their appeals. Past experience has taught the worldly wise to look with considerable suspicion upon requests for assistance which are based upon excessive sentiment rather than on sound reason and facts.

HOSPITAL ABUSE.

Another bad result of these appeals to the general public on the part of individual hospitals follows the custom of quoting in such appeals the numbers of in-patients and out-patients which have been treated in a given year, or since its foundation, at the particular institution which is applying to the generosity of the public. Such publications only serve to lead the public into making comparisons between the numbers of patients treated at the different hospitals as a criterion of their worth, while their attention is withdrawn from the more important points of efficiency and economical management. There can be scarcely an atom of doubt that a premium is thus placed upon hospital abuse. We have no hesitation in affirming that a large number of the out-patients treated at the voluntary hospitals, and especially at the special hospitals and those general hospitals where no system of inquiry into the patients' means is in use, has not the slightest right to claim or to receive medical advice and treatment at the expense of the charitable public who support the voluntary hospitals. There is another fairly numerous class of out-patient which ought to be more actively discouraged by the hospital managers than it is at present, namely, the one that attends hospital out-patient departments mainly for the social enjoyments it manages to find there, combined with an insatiable craving for bottles of medicine. A considerable section of the public is fully aware that hospital abuse does occur, and to an extent which cannot be considered unavoidable. This fact, in conjunction with some suspicions of good faith which are likely to be raised by the immoderately worded appeals of certain hospitals, may, if permitted to continue, cause a valuable, if small, section of the charitable public to take a more limited interest in the voluntary hospitals of London than it has done in the past. We should, for this reason, welcome a general decline of the yearly number of out-patients treated at the voluntary hospitals,as a sign that the managers of these institutions were more widely awake to the fact that they are entirely responsible for the large amount of hospital abuse and the consequent waste of public charity which is now allowed to occur.

Needs of the Metropolis Overlooked.

So completely is each hospital engaged in looking after its own interests that the needs of the metropolis as a whole, and of its different sections, are apt to be lost sight of. This is nowhere so apparent as in the existing distribution of the voluntary hospitals, whereby some districts—Westminster, for example—are overcrowded with hospitals, while others, such as Tottenham and Battersea, are not provided with anything approaching a sufficient complement of hospital beds.

Inferior Medical Instruction.

It is to be deplored that there is no really well-organised system of clinical instruction in London which deals with the vast mass of valuable material which is available. Not only are the Poor-law infirmaries, with their thousands of patients, closed to the student of medicine, but a student attached to one hospital cannot, with a few exceptions, attend the lectures or clinical instruction at another hospital unless he pays additional fees in each separate case. In this way, not only is he confined to a limited amount of material on which he is to base his experience and knowledge, but he is also deprived of the opportunity of selecting the best clinical teacher in each branch of the subject, and is often driven by force of circumstance to attend lectures and clinics given by men who have no natural or acquired ability whatever for imparting knowledge. Great clinical teachers are as rare as they are of priceless value, and if such a one has his lot cast in London his light is perforce concealed beneath a bushel. His power is utterly wasted except within the narrow confines of his own hospital and medical school. Once he is established as the best clinical instructor in his own hospital he has no higher triumph to win. Ability cannot be purchased cheaply, and so long as the present system continues it cannot be expected, or even hoped, that London will hold the first place in all the world as a centre of medical education. These are some of the evil results of the existing lack of hospital co-operation in London. The instances given are sufficient to demonstrate the urgency with which reform is needed, and to show how every individual in the metropolis is directly or indirectly affected. To summarise the drawbacks we have mentioned they are : Hospital malpraxis, loss of public support, competitions for public charity, indifference towards, and even encouragement of, hospital abuse, insufficient consideration for the needs of the metropolis as a whole, and a great waste of clinical material and teaching ability which places the London student of medicine at a disadvantage.

*(To be continued.)*

